# Bimetallic Core–Shell Nanoparticles of Gold and Silver via Bioinspired Polydopamine Layer as Surface-Enhanced Raman Spectroscopy (SERS) Platform

**DOI:** 10.3390/nano10040688

**Published:** 2020-04-05

**Authors:** Asli Yilmaz, Mehmet Yilmaz

**Affiliations:** 1 Department of Molecular Biology and Genetics, Ataturk University, Erzurum 25240, Turkey; asliozdilek@gmail.com; 2 East Anatolia High Technology Application and Research Center (DAYTAM), Ataturk University, Erzurum 25240, Turkey; 3 Department of Chemical Engineering, Ataturk University, Erzurum 25240, Turkey; 4 Department of Nanoscience and Nanoengineering, Ataturk University, Erzurum 25240, Turkey

**Keywords:** bimetallic core–shell nanoparticles, gold nanoparticles, polydopamine, surface-enhanced Raman spectroscopy (SERS)

## Abstract

Despite numerous attempts to fabricate the core–shell nanoparticles, novel, simple, and low-cost approaches are still required to produce these efficient nanosystems. In this study, we propose the synthesis of bimetallic core–shell nanoparticles of gold (AuNP) and silver (AgNP) nanostructures via a bioinspired polydopamine (PDOP) layer and their employment as a surface-enhanced Raman spectroscopy (SERS) platform. Herein, the PDOP layer was used as an interface between nanostructures as well as stabilizing and reducing agents for the deposition of silver ions onto the AuNPs. UV-vis absorption spectra and electron microscope images confirmed the deposition of the silver ions and the formation of core–shell nanoparticles. SERS activity tests indicated that both the PDOP thickness and silver deposition time are the dominant parameters that determine the SERS performances of the proposed core–shell system. In comparison to bare AuNPs, more than three times higher SERS signal intensity was obtained with an enhancement factor of 3.5 × 10^5^.

## 1. Introduction

Traditionally, core–shell nanoparticles are defined as composite nanomaterials constructed with inner material (cores) and outer layer material (shells), both at a nanoscale [1]. Nowadays, core–shell nanosystems have attracted increasing attraction due to their versatile compositions and structures to serve in different applications. The employment of either the same or different materials in the core or the shell provides highly functional materials and unique as well as flexible properties [2]. For instance, the report by Lim et al. indicated well-defined gold nanobridged nanogap particles through DNA molecules [3]. They could load Raman dyes to the gap (1 nm) between the gold core and gold shell. The resultant core–shell system created a highly stable and reproducible surface-enhanced Raman spectroscopy (SERS) signal. The features of core–shell nanoparticles can be modified by changing many parameters including the type of core or shell materials, the thickness of each layer, and the core to shell ratio [4,5]. In addition, core–shell nanoparticles provide distinctive properties in terms of versatility, low cost, tunability, stability, biocompatibility, and controllability [2] in comparison to the materials that are used as core or shell material. For instance, the chemical stability of some metallic nanostructures can be enhanced for practical applications via a core−shell system by creating a thin shell of silica [6]. In addition, Bian et al. showed that a thin layer of graphene deposited onto the gold nanostructures improved the biocompatibility of the core–shell system [7]. With their unique properties, core–shell nanostructures have a wide range of applications including enhanced optical devices, energy storage materials, bionanotechnology, fuel cell, tailored magnetic devices, optical devices, bioimaging systems, and catalytic processes [1,2,8,9,10,11,12].

In addition to their applications in sensing techniques and luminescence enhancements [13,14,15], specifically core−shell nanomaterials with plasmonic materials such as gold, silver, or copper provide extensive employment in surface-enhanced vibrational spectroscopies, especially in SERS [2]. Compared to single metal nanoparticles, core−shell nanoparticles serve extraordinary SERS activities due to their high potential to create unique localized surface plasmon resonance (LSPR). The optical properties of the resultant core–shell nanostructures can be manipulated due to the change in dielectric constants and the electrons in the interface.

Core–shell nanostructures as ideal SERS systems provide significant advantages in comparison to their bare counterparts [16,17,18,19]. Although there have been many studies in the field of fabrication and application of the core–shell nanoparticles, novel, simple, and low-cost approaches are still required to produce efficient nanosystems [20,21,22,23,24,25,26,27,28,29,30]. Here, for the first time, we report the novel synthesis of bimetallic core–shell nanoparticles of gold and silver via bioinspired polydopamine (PDOP) layer. For this, firstly, we prepared the citrate-stabilized gold nanoparticles (AuNPs), and then AuNPs were coated with a thin layer of PDOP simply by the oxidative polymerization of dopamine. The PDOP layer with its numerous catechol, imine, and amine groups works as a reduction agent of silver ions and stabilization of silver nanostructures [31,32]. The PDOP-coated AuNPs (AuNP@PDOP) were treated with AgNO_3_ containing a solution for the decoration of silver nanoparticles (AgNPs). The AgNPs-deposited AuNP@PDOP (AuNP@PDOP@AgNP) bimetallic core–shell nanoparticle system was tested as a SERS platform. It was shown that both PDOP thickness and silver deposition time are the dominant parameters that determine the SERS performances of the proposed core–shell system.

## 2. Experimental Methods

Synthesis and characterization of core–shell nanoparticles: Firstly, for the preparation of AuNPs as the core material, the well-known citrate reduction method with some modifications was employed similar to our earlier reports [33,34]. Briefly, in a three-neck round bottom flask, 50 mL of 1 mM aqueous solution of HAuCl_4_ was heated to boiling under vigorous stirring. Afterward, the gold ions were reduced by the addition of the proper amount of (0.1 M, 1.65 mL) trisodium citrate solution to the boiling mixture. The heater was turned off, and the AuNPs suspension was cooled to room temperature. For the purification, the resultant ruby-red colored AuNPs were centrifuged at 14,000 rpm for 20 min and washed with deionized (DI) water and re-dispersed in water. A thin conformal PDOP layer was coated onto the core surface with an oxidative polymerization of dopamine (2 mg/mL, pH: 10.5) in Tris buffer for different reaction times (30, 60, and 180 min). In our previous reports, we depicted that the PDOP thickness can be manipulated simply by tuning the polymerization time [35,36,37,38,39]. The resultant AuNP@PDOP nanoparticles were purified by using centrifugation (14,000 rpm, 20 min). For the fabrication of the core–shell nanostructure, AuNP@PDOP nanoparticles were immersed into the silver nitrate solution for different reduction times (10 mM AgNO_3_, 30, and 60 min). Here, the PDOP layer with its numerous catechol, imine, and amine groups works in both the reduction of silver ions and stabilization of silver nanostructures. All the nanoparticle synthesis steps were monitored by the employment of a Shimadzu UV-3600 Plus ultraviolet-visible (UV–vis) UV–vis–near-infrared (IR) spectrophotometer. In addition, the morphology of NP systems was analyzed on a transmission electron microscope (TEM, Hitachi HighTech HT7700, and FEI Talos F200X) with an energy-dispersive X-ray spectroscopy spectrometer (EDAX). With these procedures, the obtained AuNP@PDOP@AgNP nanoparticles can be used in different applications, including the SERS platform. All chemicals with ACS grade purity were supplied from Sigma-Aldrich.

SERS measurements: To determine the SERS activity of different nanoparticle systems (AuNP, AuNP@PDOP, and AuNP@PDOP@AgNP), a WITech alpha 300R Micro Raman Microscopy system was employed in the following experimental conditions: 785 nm laser source, ×50 objective, 20 s acquisition time, 1 µm spot size, and 10 mW laser power. All Raman measurements were performed in the range of 200–2000 cm^−1^, and baseline correction was applied to all spectra by employing OriginPro 2019 software. Methylene blue (MB) was selected as the Raman active dye in SERS measurements because of its high Raman activity, well-defined Raman bands, and relatively high stability. In a typical procedure, 900 µL of nanoparticle suspension and 100 µL of stock MB solution (1 × 10^−4^ M) were mixed to obtain the suspension in a desired MB final concentration (1 × 10^−5^ M). Then, 5 µL of the suspension was dropped onto the aluminum film and kept in a hood until dry. The drying procedure led to coin-shaped Raman sample sizes of 4 ± 1 mm onto the surface. To eliminate the reproducibility problems due to the coffee ring effect, we collected at least 20 spectra from a different point of the sample and spectra with average intensity (calculated according to the prominent peak at 1621 cm^−1^) was selected and given in the manuscript.

## 3. Results and Discussion

### 3.1. Morphological and Elemental Analysis of NP Systems

**Figure 1:**
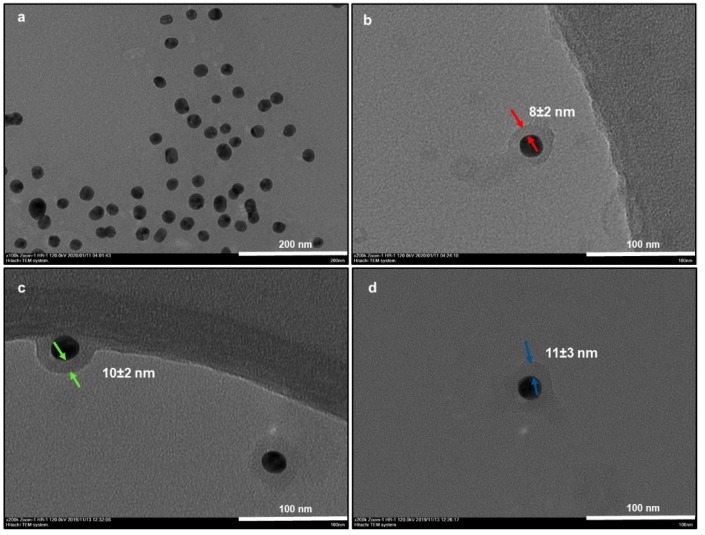
TEM images ((**a**): bare AuNP, (**b**): AuNP@30PDOP, (**c**): AuNP@60PDOP, and (**d**): AuNP@180PDOP) of AuNP and AuNP@PDOP for different polydopamine (PDOP) deposition times (numbers indicate deposition times in min). AuNP@PDOP: PDOP-coated gold nanoparticles immersed into silver nitrate solution for different reduction times (10 mM AgNO_3_, 30, 60, and 180 min).

**Figure 2:**
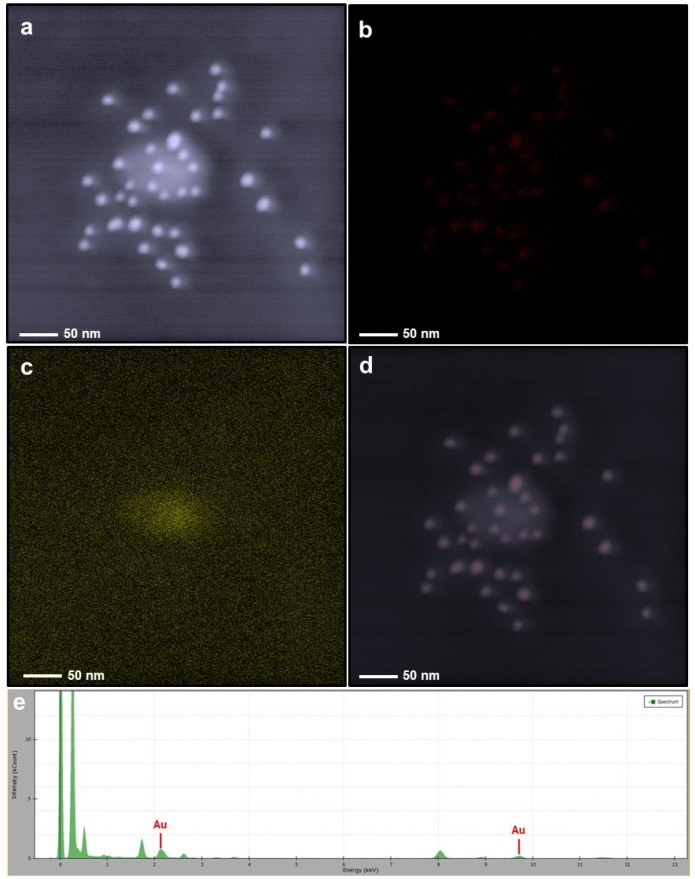
Elemental mapping and energy-dispersive X-ray spectroscopy (EDX) spectrum of the AuNP@30PDOP NP system. Electron micrograph region (**a**), distribution of gold elemental mapping (**b**), distribution of carbon elemental mapping (**c**), overlay of gold and carbon, and (**d**) relevant energy-dispersive X-ray spectroscopy spectrometer (EDAX) spectrum (**e**).

In the first part, we performed some TEM analysis with elemental mapping and the EDAX spectrum. TEM images of bare AuNPs indicated an efficient synthesis of spherical metallic nanoparticles with an average size of 18 nm (Figure 1a). After an oxidative polymerization of dopamine, the presence of the PDOP layer onto the AuNPs was confirmed from TEM images for different PDOP deposition times (Figure 1b–d). Similar to our previous reports, for these polymerization times (30, 60, and 180 min), we observed that the thickness of the PDOP layer ranged from 8 ± 2 to 11 ± 3 nm [33,35,36,38,39]. Here, we observed that the thickness of the PDOP could be easily manipulated by tuning the deposition time. In addition, a representative elemental mapping image and EDAX analysis of the AuNP@30PDOP NP system showed the emergence of the PDOP layer after dopamine oxidative polymerization (Figure 2). The relatively higher content of C element around the AuNPs was observed, indicating the deposition of the PDOP layer. EDAX spectra (Figure 2e) furtherly confirmed the presence of metallic NPs. The other peaks in the spectra were assigned to the copper and carbon content of the TEM grid.

**Figure 3:**
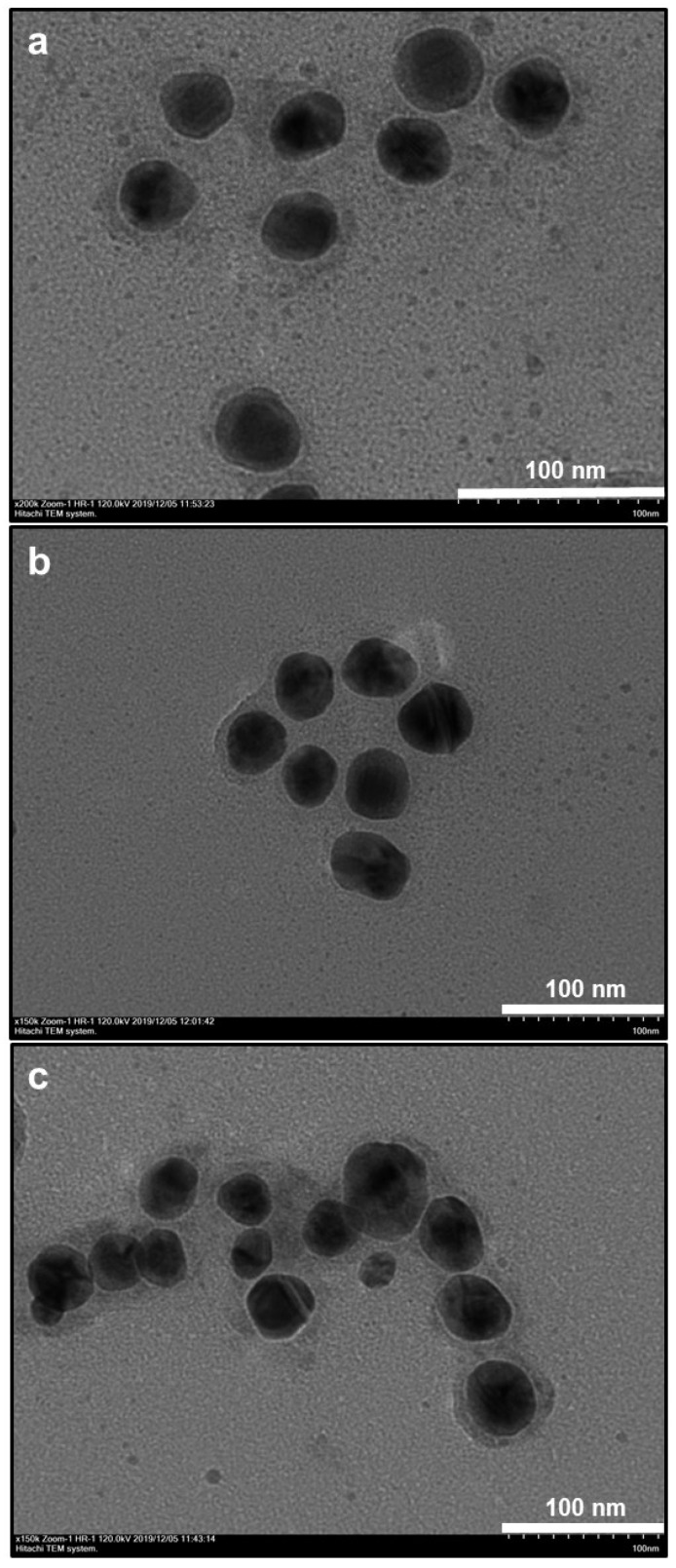
Effect of PDOP polymerization time (i.e., PDOP thickness) on core–shell NPs morphology for 30 min of silver reduction time. TEM images ((**a**): AuNP@30PDOP@30AgNP, (**b**): AuNP@60PDOP@30AgNP, and (**c**): AuNP@180PDOP@30AgNP) of a bimetallic core–shell NP system for different PDOP deposition times (numbers indicate deposition times in min).

**Figure 4:**
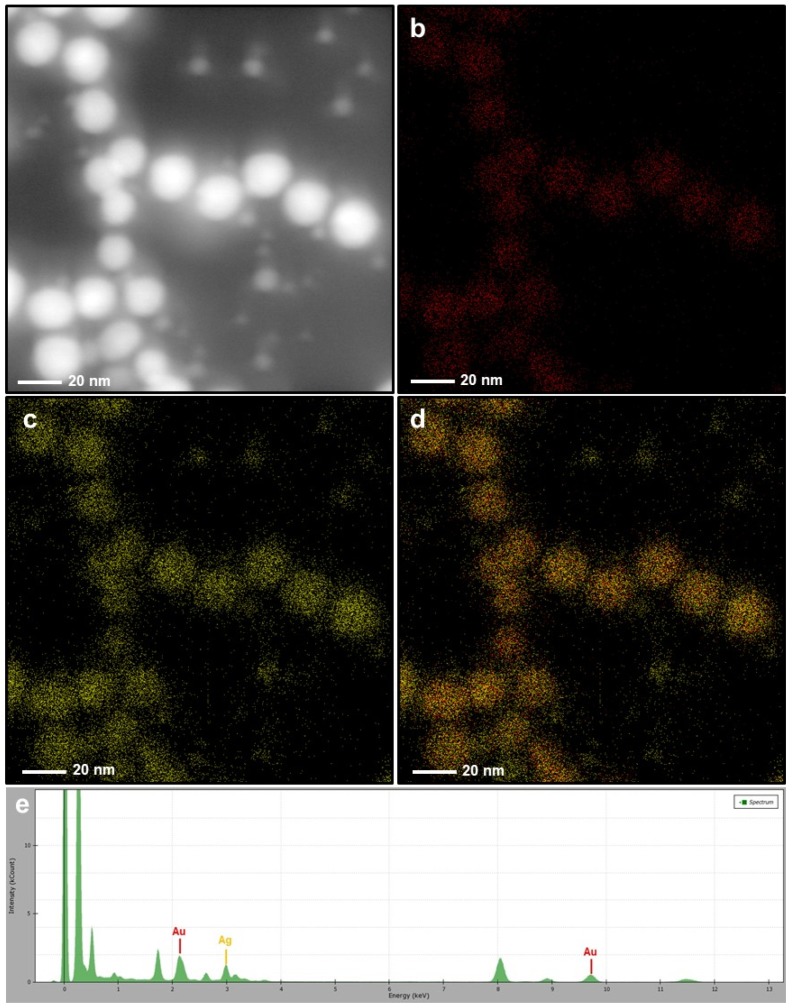
Elemental mapping and EDAX spectrum of the AuNP@30PDOP@30AgNP system. Electron micrograph region (**a**), distribution of gold elemental mapping (**b**), distribution of silver elemental mapping (**c**), overlay of gold and silver (**d**), and relevant EDX spectrum (**e**).

**Figure 5:**
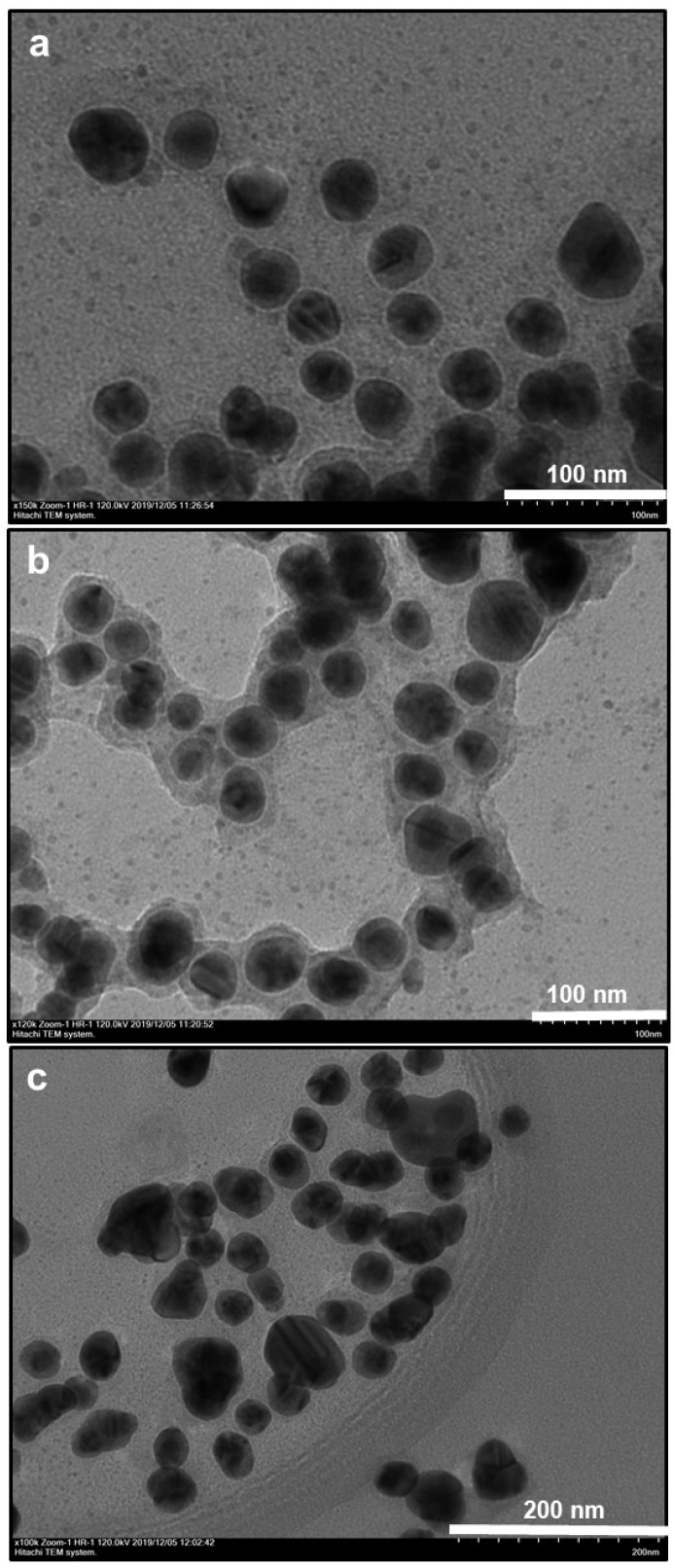
Effect of PDOP polymerization time (i.e., PDOP thickness) on core–shell NPs morphology for 60 min of silver reduction time. TEM images ((**a**): AuNP@30PDOP@60AgNP, (**b**): AuNP@60PDOP@60AgNP, and (**c**): AuNP@180PDOP@60AgNP) of bimetallic core–shell NP system for different PDOP deposition times (numbers indicate deposition times in min).

**Figure 6:**
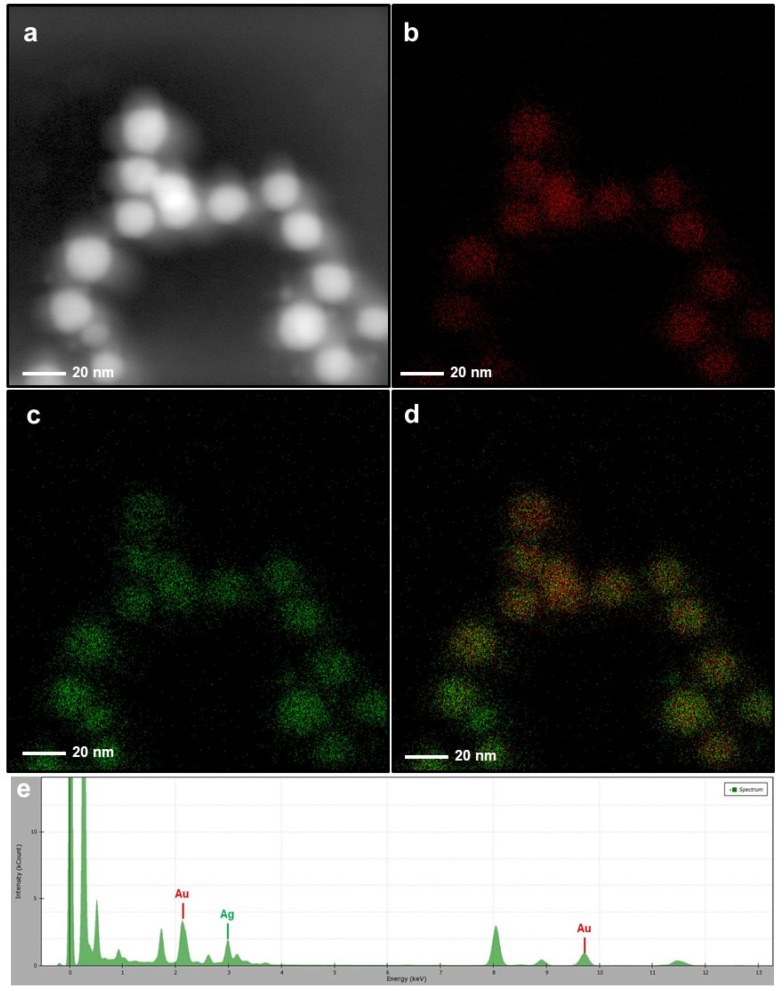
Elemental mapping and EDAX spectrum of the AuNP@30PDOP@60AgNP system. Electron micrograph region (**a**), distribution of gold elemental mapping (**b**), distribution of silver elemental mapping (**c**), overlay of gold and silver (**d**), and relevant EDX spectrum (**e**).

After immersion of the AuNP@PDOP nanoparticles into the AgNO_3_ solution, the dramatic change in the structure of nanoparticles was detected. For the case of 30 min of silver reduction time (Figure 3), a thin layer of silver deposition was detected as the shell material. As the PDOP thickness was increased, the thickness of the silver shell was increased accordingly. The thickness of the silver shell ranged from 7 ± 3 to 9 ± 5 nm (Figure 3). This result is somewhat expected. The higher polymerization time of dopamine led to a higher thickness of PDOP, which is essential for the reduction of silver ions. PDOP, having vast functional groups such as catechol, amine, and imine, can reduce metal ions as metallic nanoparticles without the employment of any reductive agent or metallic seed particles [31,32,33,35,38,39]. Besides, PDOP can stably adsorb metallic nanoparticles onto its surface for a long time. For further confirmation, we also collected some elemental mapping analysis with EDAX spectrum. This analysis of the AuNP@30PDOP@30AgNP system was summarized in Figure 4. Elemental mapping indicated that almost all NP systems (>95%, by analyzing at least 100 NPs for each case) were in the form of a core–shell structure. In addition to gold, the presence of the silver as the shell material was also confirmed in the EDAX spectra (Figure 4e). Similar to the PDOP deposition time, the increase in the silver deposition time led to a remarkable increase in the amount of deposited silver content and thickness. The effect of 60 min of silver reduction time for different PDOP polymerization time on core–shell NPs morphology was summarized in Figure 5 through representative TEM images. Our detailed TEM analysis revealed that the thickness of the silver shell ranged from 9 ± 3 to 12 ± 5 nm. To further confirm this issue, we also performed elemental mapping data and EDX spectra for the AuNP@30PDOP@60AgNP system (Figure 6). In comparison to AuNP@30PDOP@30AgNP (Figure 4), we noticed the higher deposition of the silver through the higher distribution of silver in elemental mapping and its relatively higher peak intensity in the EDAX spectrum (Figure 6). In light of detailed TEM and an elemental analysis of NP systems, we can conclude that core–shell NP systems were efficiently prepared via the proposed PDOP-based approach and that both PDOP thickness and silver reduction time have a significant effect on the resultant NP system.

### 3.2. UV-Vis Spectroscopy Analysis

**Figure 7:**
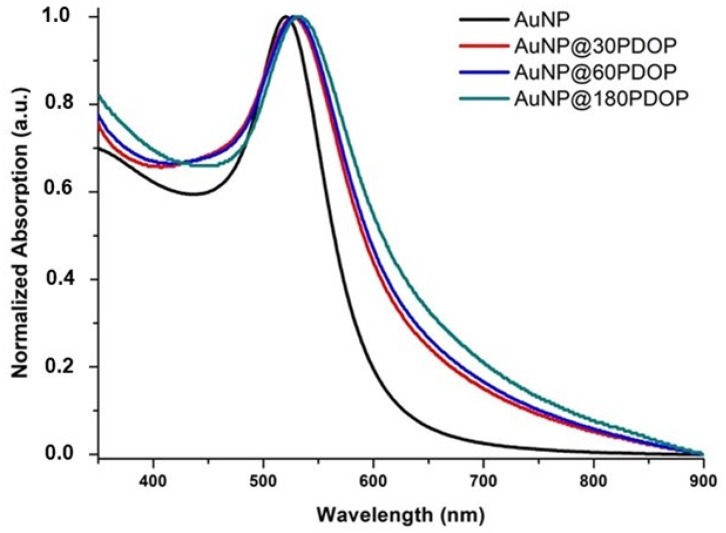
UV-vis absorption spectra of AuNP and AuNP@PDOP for different PDOP deposition times (numbers indicate deposition times in min).

The efficient synthesis of AuNPs was confirmed from ruby-red color by the naked eye and UV-vis absorption spectra (Figure 7). AuNPs showed sharp absorption maxima at 521 nm, which can be attributed to the presence of spherical nanoparticles with an average size of 18 nm [33,40,41]. After the deposition of the PDOP layer onto the AuNPs, a remarkable redshift (7–11 nm) was detected in the absorption maxima of the AuNP@PDOP nanoparticles (see Table 1). This phenomenon is due to the change in dielectric constants because of the deposition of a thin PDOP layer. When the PDOP deposition times—in other words, the PDOP thickness—was increased, the degree of the redshift was increased accordingly (Figure 7).

**Figure 8:**
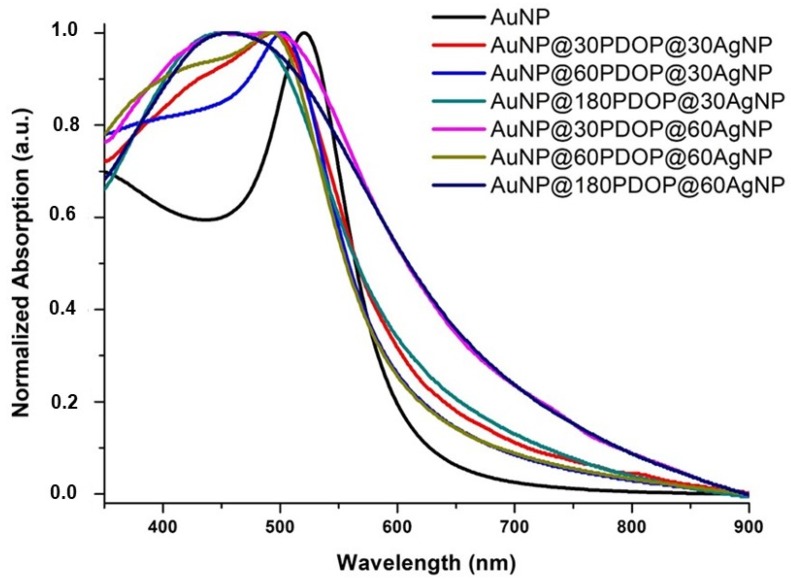
UV-vis absorption spectra of AuNP and AuNP@PDOP@AgNP for different PDOP and silver deposition times (numbers indicate deposition times in min).

After immersion of the AuNP@PDOP nanoparticles into the AgNO_3_ solution, the distinctive change was detected in the color of the NP suspension. This observation was furtherly confirmed by the UV-vis absorption spectra, which were given in Figure 8. After the reduction of silver ions, an intense blue shift with remarkably widened spectra was observed for each case of the AuNP@PDOP nanoparticles (see Table 1) [42,43]. The degree of the blue shift is highly correlated with the amount of the reduced silver ions and the resultant silver shell thickness [44,45,46,47,48]. The broadening in SPR characteristics of the core–shell metallic NP systems can be attributed to many independent variables including the composition of core or shell material and their morphology and thickness or diameter [49,50]. It must be also noted AuNP as the core material preserved its SPR properties, resulting in a broader absorption peak after silver reduction [42]. This obvious observation shows the possibility of manipulating SPR properties by the employment of core–shell nanostructures. In our case, when both PDOP thickness and silver deposition time was increased, the degree of the blue shift was increased accordingly. The longer deposition time of silver ions also results in their higher reduction as metallic nanoparticles. By combining TEM and UV-vis absorption spectra, we can conclude that both the PDOP deposition time and silver reduction time have a huge impact on the fabrication of core–shell nanostructures.

### 3.3. SERS Analysis

**Figure 9:**
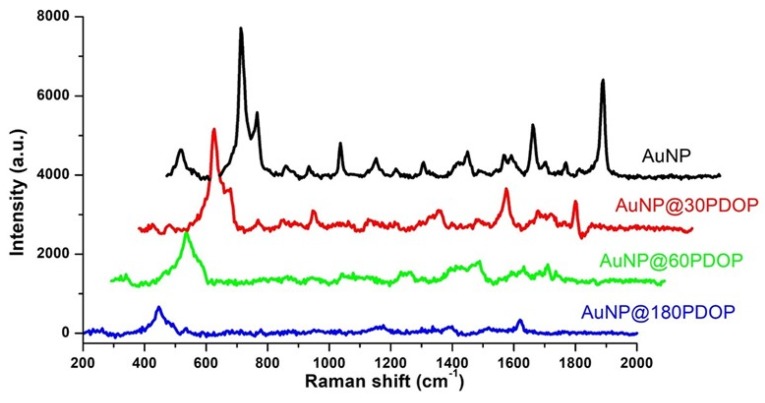
Representative Raman measurements of AuNP and AuNP@PDOP for different PDOP deposition times (numbers indicate deposition times in min). The final concentration of methylene blue (MB) for Raman measurement is 10^−5^ M.

After the fabrication of each NP system, we used these nanoparticles as a SERS platform. For the case of bare AuNPs, we collected precise SERS spectra with a high signal-to-noise ratio (Figure 9). No signal was detected in the absence of MB (data not shown here). Similar to our previous reports [33,34,35,36,39,51,52,53], all the Raman bands were assigned to MB. Some prominent peaks in the spectra of MB were summarized as follows: the C–N–C skeletal deformation mode (δ(C–N–C)) at 450 cm^−1^, the ring stretch (ϒ(C–C)) at 1621 cm^−1^; symmetric and asymmetric C–N stretches (ϒ_sym_(C–N) and ϒ_asym_(C–N)) at 1393 and 1435 cm^−1^, respectively. However, the presence of the PDOP layer led to a remarkable decrease in SERS signals in comparison to bare AuNPs (Figure 9). This result is highly expected. Here, the PDOP layer hindered the interaction of the dye molecules and plasmonic nanostructures to facilitate electromagnetic enhancement [33,39]. As the PDOP deposition time—that is to say, the PDOP thickness—increased, the decrease in SERS signal was more remarkable accordingly.

To quantify the SERS activity of the platforms, we also calculated the enhancement factors (EFs) through Equation (1) by employing the peak intensity of MB at 1621 cm^−1^.

EF = (N_bulk_ × I_SERS Platform_)/(N_SERS Platform_ × I_bulk_) (1)

Herein, I_bulk_ and I_SERS Platform_ are the Raman peak intensities obtained from bulk MB and the adsorbed MB on the SERS platform, respectively, and N_bulk_ and N_SERS Platform_ are the calculated number of MB molecules for the reference sample and SERS platform, respectively. EF values for each SERS platform are depicted in the last column in Table 1.

In an ideal SERS system, the well-controlled distance in nm scale between two plasmonic nanostructures is essential for the enhancement in the electric field distribution. In our proposed system, this distance can be controlled with highly acceptable sensitivity through the thickness of the PDOP layer. Here, PDOP would be used as an interphase between AuNP and AgNP as two plasmonic nanostructures. These two nanostructures with a well-controlled distance would create robust SERS activity. In this study, we aimed to enhance the SERS activity of AuNPs by combining AuNPs with AgNPs and obtaining core–shell bimetallic nanostructures through the bio-inspired PDOP layer.

**Figure 10:**
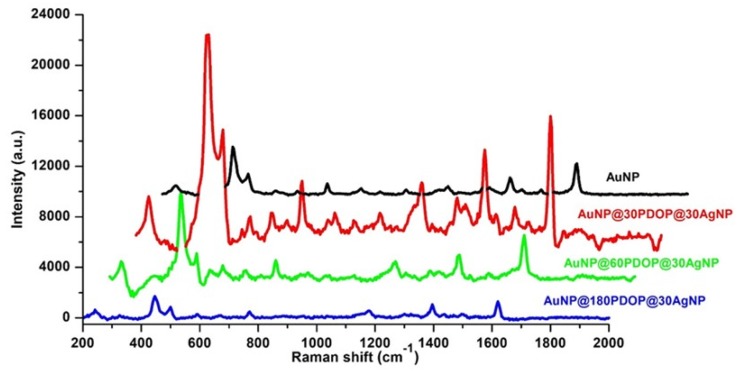
Effect of PDOP polymerization time (i.e., PDOP thickness) on SERS effect for 30 min of silver reduction time. Representative Raman measurements of the bimetallic core–shell NP system for different PDOP deposition times (numbers indicate deposition times in min). The final concentration of MB for Raman measurement is 10^−5^ M.

After silver deposition, the resultant core–shell platforms were used as SERS systems. Figure 10 summarizes the SERS activity of the platforms with representative spectra for different PDOP thicknesses (30, 60, and 180 min) and fixed silver deposition time (30 min). For the case of AuNP@30PDOP@30AgNP, the remarkable increase was detected in comparison to the bare AuNP system (3.5 × 10^5^ EF, 3.5 times higher than bare AuNP, see Table 1). The increase in SERS signal basically can be attributed to the synergistic action between AuNP and AgNP as core and shell materials, respectively. It was shown by some theoretical and experimental observations that plasmonic nanostructures having proximity in a nm scale can create enhanced electrical field distribution and be used as an ideal SERS platform [34]. The model developed by Garcia-Vidal and Pendry has depicted that for the case of isolated particles, the average enhancement is about 10^3^ [54]. However, when the plasmonic nanostructures are almost in direct contact, the average enhancement reaches its maximum value with 10^6^. In light of this data, it is highly reasonable to expect SERS enhancement for our proposed core–shell bimetallic system. However, a further increase in PDOP deposition times (i.e., PDOP thickness) resulted in lower SERS activity. From these data, we can conclude that 30 min of PDOP deposition time (i.e., 8 ± 2 nm of PDOP thickness) is the optimum value to obtain the highest SERS performance.

**Figure 11:**
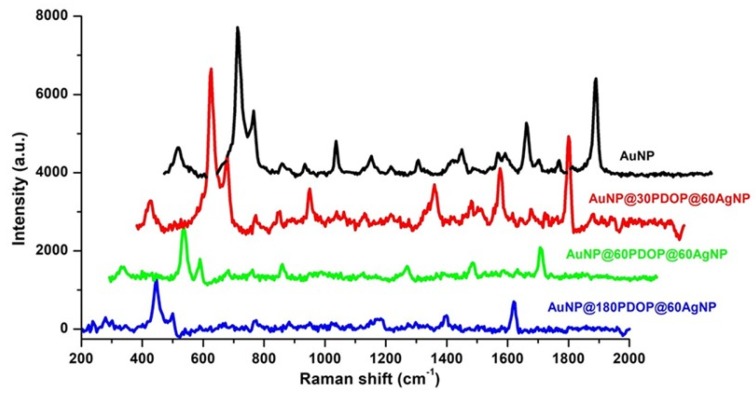
Effect of PDOP polymerization time (i.e., PDOP thickness) on SERS effect for 60 min of silver reduction time. Representative Raman measurements of the bimetallic core–shell NP system for different PDOP deposition times (numbers indicate deposition times in min). The final concentration of MB for Raman measurement is 10^−5^ M.

When the silver reduction time was increased to 60 min (Figure 11), almost the same (for the case of AuNP@30PDOP@60AgNP) or lower (for the cases of AuNP@60PDOP@60AgNP and AuNP@180PDOP@60AgNP) Raman signal intensities were obtained compared with bare AuNPs. This issue can be attributed to the higher deposition of silver on the AuNPs and the probable loss of synergistic action between AuNP and AgNP. In line with UV-vis absorption data (Figure 8 and Table 1) and TEM images (Figure 5), as the silver reduction time increased, the thickness of the silver as the shell material increased accordingly. It is clear that the higher reduction time of silver ions led to a higher thickness of the silver shell. The decrease in SERS efficiency can be attributed to the loss of the LSPR effect between the Au core and Ag shell [3]. In our case, the distance between these two plasmonic metals, which can create hotspots for enhanced SERS activity, is governed by the PDOP layer. However, it seems that the over deposition of silver ions led to the loss of this nanogap and resultant LSPR. Therefore, we obtained lower SERS activity for the cases of higher silver deposition. This phenomenon requires further experimental and theoretical research, which will be the subject of the future project. However, from these data, we can conclude that both PDOP thickness and silver deposition time are the dominant parameters that determine the SERS activity of the AuNP@PDOP@AgNP core–shell system.

## 4. Conclusions

In this study, we efficiently prepared bimetallic core–shell nanostructures of gold and silver through a thin layer of PDOP as an interface. Here, PDOP also served as stabilizing and reducing agents for the reduction of silver ions and adsorption of silver nanostructures. After the addition of silver onto the AuNPs, remarkable enhancement was detected in the SERS activity of the proposed system under the optimized experimental conditions. It was detected that both PDOP thickness and silver deposition time have a certain effect on the SERS activity of the AuNP@PDOP@AgNP core–shell system. In the future, with the bio-inspired PDOP layer, this novel, simple, and low-cost approach can be applied not only to the SERS test but also to catalytic applications and cytotoxicity tests.

## Figures and Tables

**Table 1:** Absorption maxima and calculated enhancement factor (EF) values for each surface-enhanced Raman spectroscopy (SERS) system.

SERS System	Absorption Maxima (nm)	SERS Enhancement Factor (EF)
AuNP	521	1.1 × 10^5^
AuNP@30PDOP	528	0.25 × 10^5^
AuNP@60PDOP	529	0.05 × 10^5^
AuNP@180PDOP	532	0.04 × 10^5^
AuNP@30PDOP@30AgNP	496	3.5 × 10^5^
AuNP@60PDOP@30AgNP	501	1.25 × 10^5^
AuNP@180PDOP@30AgNP	455	0.8 × 10^5^
AuNP@30PDOP@60AgNP	490	1 × 10^5^
AuNP@60PDOP@60AgNP	492	0.38 × 10^5^
AuNP@180PDOP@60AgNP	455	0.3 × 10^5^
